# A quality improvement project to increase self-administration of medicines in an acute hospital

**DOI:** 10.1093/intqhc/mzy035

**Published:** 2018-03-24

**Authors:** S Garfield, H Bell, C Nathan, S Randall, F Husson, C Boucher, A Taylor, J Lloyd, A Backhouse, L Ritchie, B D Franklin

**Affiliations:** 1 Charing Cross Hospital, Imperial College Healthcare NHS Trust, Fulham Palace Rd, Hammersmith, London, UK; 2 Department of Practice and Policy, UCL School of Pharmacy, 29-39 Brunswick Square, Bloomsbury, London, UK

**Keywords:** Self-administration, quality improvement, medicines, hospital, patient involvement

## Abstract

**Quality problem or issue:**

A patient survey found significantly fewer patients reported they had self-administered their medicines while in hospital (20% of 100 patients) than reported that they would like to (44% of 100). We aimed to make self-administration more easily available to patients who wanted it.

**Initial assessment:**

We conducted a failure, modes and effects analysis, collected baseline data on four wards and carried out observations.

**Choice of solution:**

Our initial assessment suggested that the main areas we should focus on were raising patient awareness of self-administration, changing the patient assessment process and creating a storage solution for medicines being self-administered. We developed new patient information leaflets and posters and a doctor’s assessment form using Plan–Do–Study–Act cycles. We developed initial designs for a storage solution.

**Implementation:**

We piloted the new materials on three wards; the fourth withdrew due to staff shortages.

**Evaluation:**

Following collection of baseline data, we continued to collect weekly data. We found that the proportion of patients who wished to self-administer who reported that they were able to do so, significantly increased from 41% (of 155 patients) to 66% (of 118 patients) during the study, despite a period when the hospital was over capacity.

**Lessons learned:**

Raising and maintaining healthcare professionals’ awareness of self-administration can greatly increase the proportion of patients who wish to self-administer who actually do so. Healthcare professionals prefer multi-disciplinary input into the assessment process.

## Quality problem or issue

The importance of involving patients with their medication is increasingly recognised [1], including in enhancing safety [2, 3]. Such involvement can increase satisfaction, improve health outcomes and reduce the likelihood of avoidable harm [4]. There is evidence for benefits of self-administration of prescribed medication in the hospital setting. Systematic reviews suggest some significant improvements in patient satisfaction, knowledge and compliance, although findings have been inconclusive [5, 6]. More recently, it was found that significantly fewer doses were omitted in patients self-administering in hospital than when medicines were administered by nurses [7]. The same study also found that patients not self-administering were more likely to have omissions of critical medicines with greater potential for harm.

Patient and public input at our organisation, a London teaching hospital, suggested increasing self-administration as a means of building relationships between inpatients and healthcare professionals, maintaining patients’ independence and providing a seamless transfer to home. A patient survey also found that significantly fewer patients reported that they had self-administered their own medicines while in hospital (20% of 100 patients) than reported that they would like to have done (44% of 100 patients, *p* ≤ 0.001) [8]. This is despite the fact that 76% of 100 healthcare professionals stated that they would support patients self-administering medicines in hospital [8].

Further research at our organisation [9] revealed specific barriers to self-administration of medication, including patients not being aware of the option of self-administration and the assessment process being perceived by staff and patients to be very arduous. We therefore carried out a quality improvement (QI) project to overcome those challenges. Our improvement aim was to increase the proportion of patients who wished to self-administer who were able to do so. Our improvement team comprised nursing and pharmacy staff, lay partners and quality improvement leads. Medical staff were represented in the wider advisory group. The project was approved as a service evaluation within the hospital organisation and is reported according to Standards for QUality Improvement Reporting Excellence (SQUIRE) guidelines [10]. It lasted for 1 year.

## Initial assessment

First, we conducted a failure modes and effects analysis [11]. We held two sessions and invited further contributions by e-mail. In total, we had input from three pharmacists, one medical consultant, one ward manager, one specialist diabetes nurse and four members of the public. We sought descriptive rather than quantitative outputs [11]. The main risks identified were (i) patients without consistent capacity being allowed to self-administer, leading to missed doses or poor adherence and (ii) patients who were suitable for self-administration not being allowed to do so, leading to delayed doses for patients who need medications at specific times and increased risk of poor administration of medication after discharge.

Second, we collected baseline data on four study wards comprising an acute medical ward, an endocrinology and rheumatology ward, a surgical ward and a private ward. We collected data to inform the following measures:

- Proportion of all patients recorded as self-administering medication.
- Proportion of all patients reporting being aware of the self-administration option.
- Proportion of patients who wished to self-administer their medication who reported that they had done so.
- Proportion of patients self-administering medication who had the correct assessment paperwork completed.
- Proportion of patients reporting that they had self-administered who were recorded as doing so.

Using a patient survey administered by a member of the project team, we collected data on each of these measures for 12 weeks to establish a baseline. We visited each ward weekly and for all patients deemed well enough we asked about their knowledge of the option of self-administration, whether they would like to self-administer their own medicines in hospital and whether they were currently doing so, using 5-point Likert scales. We also collected point prevalence data on the proportion of patients recorded as self-administering and the proportion self-administering who had the correct paperwork completed at each ward visit (see Appendix 1 for data collection tool). We plotted the results using statistical process control P-charts [12].

Across the 12 weeks, we found that a mean of

- 7% of 900 patients were recorded as self-administering medication,
- 37% of 329 patients reported being aware of the self-administration option,
- 41% of 115 patients who wished to self-administer reported self-administering any of their medicines,
- 31% of 55 patients who reported self-administering were recorded as such on the electronic prescribing system.

No self-administering patients had the correct assessment paperwork completed.

Third, we carried out observations on a ward specialising in endocrinology and rheumatology. We observed two doctor's ward rounds, a nurses' drug administration round and a pharmacists' visit to the ward.

**Figure 1:**
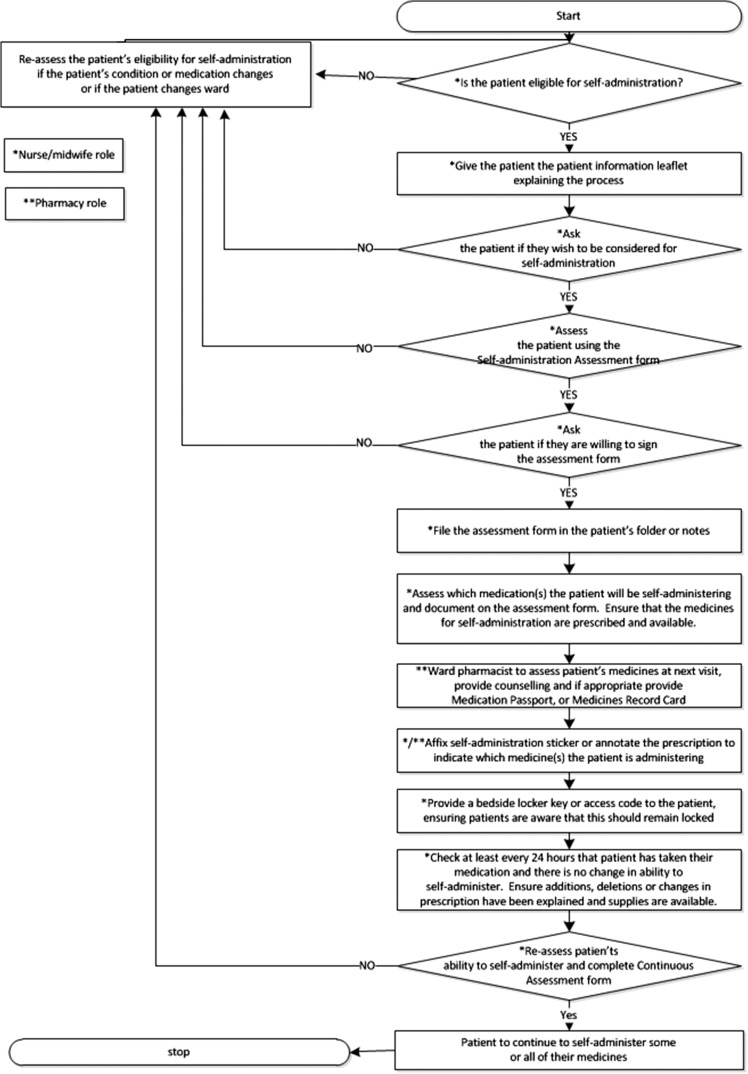
Process map of the self-administration process as outlined in the self-administration policy.

**Figure 2:**
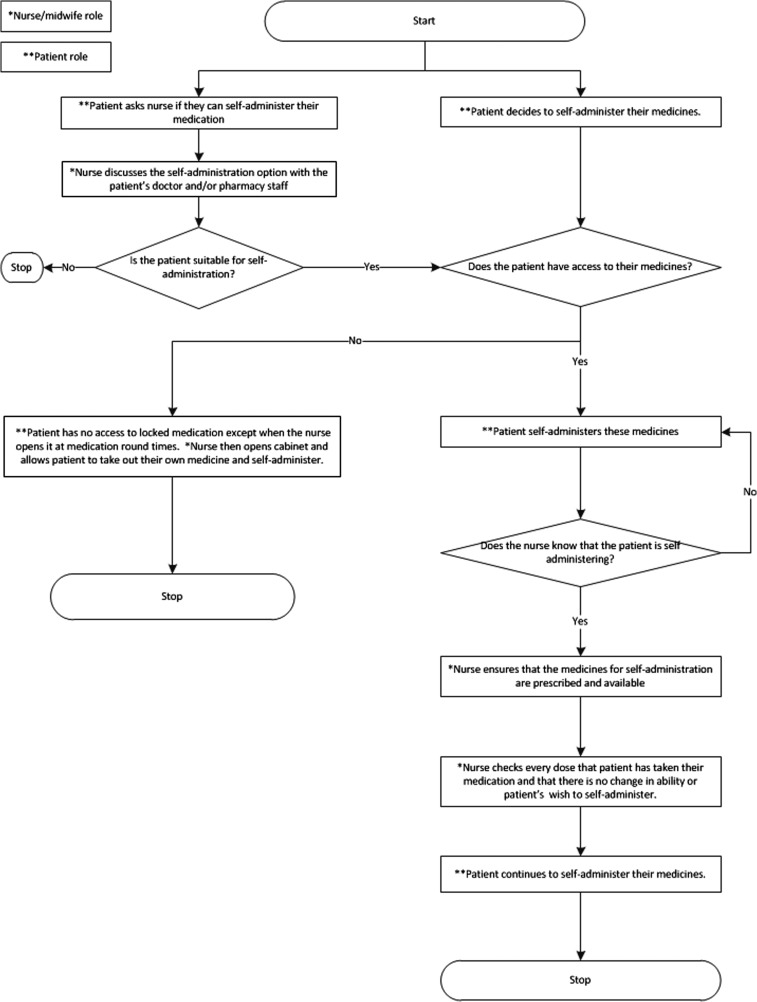
Process map of the most common self-administration processes observed.

We produced process maps of the self-administration process according to both the hospital self-administration policy and the actual self-administration process that we observed (Figs 1 and 2) and found many discrepancies between them. According to the self-administration policy, all patients should be assessed for eligibility for self-administration; however, in practice, only a small of number of patients were considered, usually on patients’ own initiation. The policy also specified that patients should be formally assessed by nursing staff prior to self-administration, but in practice we found that nurses either carried out no assessment or had informal discussions with medical or pharmacy staff. Finally, according to the policy, patients who were self-administering should have been provided with a key or access code for their bedside medicines locker but in practice no patients were provided with these. Either patients kept their medication unlocked by their bedside or nurses opened the cabinet during the medication round and allowed the patient to take out their medicine to administer.

## Choice of solution

**Figure 3:**
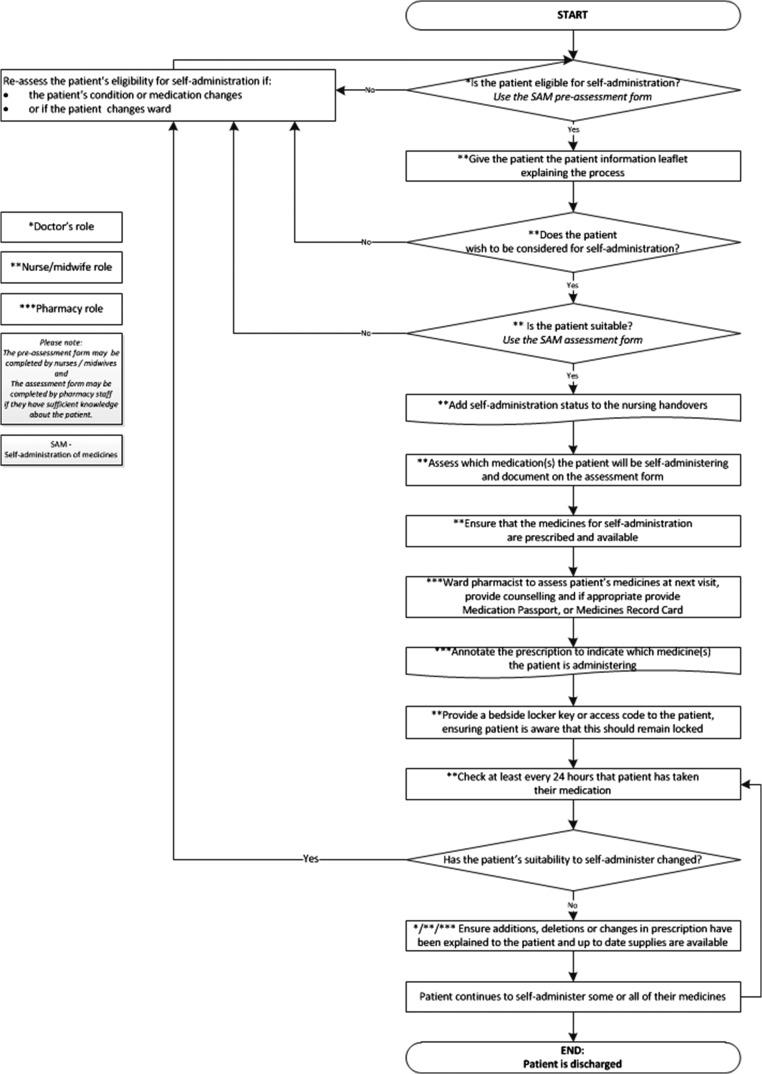
Process map of the new proposed self-administration process.

Initial findings suggested that three of the key areas to work on were patient awareness of the self-administration option, the assessment process and storage of medicines for self-administration. We attended a QI day at the hospital organisation to take each of these forward. The project team met with members of the public, healthcare professionals and designers to work collaboratively to create solutions. Participants visited hospital wards and discussed potential solutions which were then developed and tested using a series of Plan–Do–Study–Act (PDSA) cycles [13]. A process map for a new self-administration process was developed to reflect these changes (Fig. 3).

### Patient awareness of the self-administration option

There was previously a patient information leaflet available in the hospital self-administration policy. However, it was concluded that this needed to be less wordy and more attractive to patients. At the QI day, participants discussed essential information that patients would need and drafted wording for the leaflet to reflect this. A designer provided initial ideas for making it look more attractive.

In addition, participants at the QI day suggested development of a poster to be put up on wards to alert patients to the option of self-administration; wording for this was also drafted.

Following the QI day, these materials were developed over four PDSA cycles; these included input from patients on one ward, a designer, the project’s patient and clinical engagement group, and the communications team at the hospital organisation. The final posters and leaflets were then printed and tested with patients on the study wards. Feedback was positive; minor final alterations were made in response (see Appendices 2 and 3 for final leaflets and posters).

### Development of a doctor's assessment form

A senior doctor at the QI day reported that, in his experience, nurses did not want to take the responsibility for assessing patients for self-administration and that where he had suggested self-administration for specific patients the nurses had asked him to sign to indicate that he was happy for the patient to self-administer. This led to a discussion about whether doctors should be responsible for assessment rather than nurses. A nurse expressed the view that nurses should also be involved as they potentially spent more time with patients. From these discussions, the idea of a joint assessment process was developed, whereby a doctor would conduct an initial assessment and a nurse would then check that there were no issues that made patients unsuitable for self-administration. The QI day participants drafted a ‘tick-box’ initial assessment form for the doctors to complete. They also suggested that this be incorporated into the electronic prescribing system recently implemented in the hospital, rather than being a separate paper-based process.

Following the QI day, the assessment was developed over two PDSA cycles. The project lead discussed the doctors’ assessment with a medical consultant working on one of the study wards, who was strongly in favour of this approach and suggested an alteration to the form to allow for change in patients’ ability to self-administer during a hospital admission. The assessment was, therefore, revised and taken to the project team who suggested one further amendment which was made.

### Storage of medicines for self-administration

A furniture designer produced some designs for medicines cabinets that would be more accessible to patients self-administering than the current cabinets. These generated much interest but it was not possible to develop these further within the time and resources available.

## Implementation

### Patient leaflets and posters

The new leaflets and posters were implemented on three of the four study wards. The fourth declined to implement any interventions due to staff shortages. The project lead met with senior nurses on the other three wards to discuss implementation. All agreed to display the new leaflets and posters. A lay partner then visited the ward with the project lead to check the positioning of the leaflets and posters and made some suggestions to increase the numbers of posters and places where leaflets were displayed, which were then implemented.

### Doctors’ assessment form

The project lead had several meetings with the hospital electronic prescribing team to discuss incorporation of the doctors’ assessment form into the electronic system. It was concluded that the only way to test this on a small number of wards, rather than organisation-wide, was for each doctor to set the assessment up on their individual profile. One registrar set this up and piloted the form; he reported it to be straightforward to use. We put posters in the doctors’ offices on the study wards inviting other doctors to pilot the form but no further piloting took place. The potential possibility of pharmacists piloting the form was also suggested at the project’s patient and clinical engagement group meeting. However, ward pharmacists did not feel that they had capacity to incorporate this into their practice.

## Evaluation

We carried out both quantitative and qualitative data collection and analysis.

For quantitative analysis, we continued collecting weekly data and plotting it on P-charts using the same metrics as for baseline data. We collected data for 43 weeks in total, including the baseline, to evaluate whether the series of interventions (with the final changes implemented at week 37) had an effect. We analysed the data using standard statistical process control rules [13] to identify significant changes in the data.

**Figure 4:**
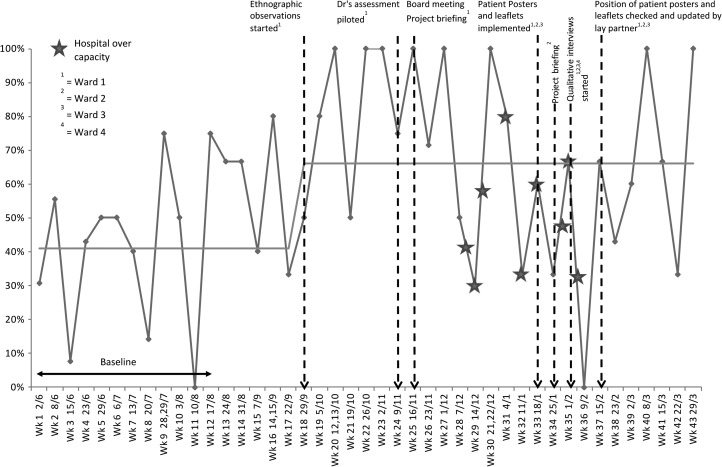
Proportion of patients who wished to self-administer their medication who reported that they had done so (data collected May 2016–March 2017).

We found that the proportion of patients who wished to self-administer who reported that they were able to do so significantly increased from 41% of 115 patients to 66% of 118 patients during the study, from baseline (Fig. 4) despite the hospital being over capacity at one period during the study. There was also a small but significant increase in patients being recorded as self-administering.

**Figure 5:**
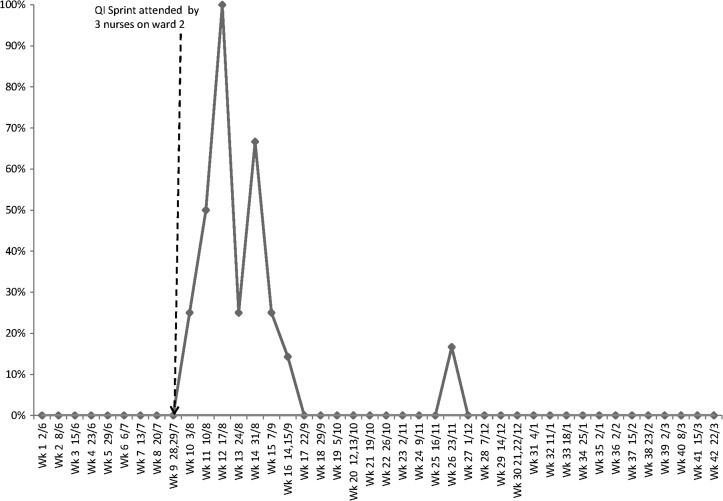
Proportion of patients self-administering who had the correct paperwork completed (data collected May 2016–March 2017).

We found that following the QI day, which was attended by three nurses who worked on one of the study wards, the number of patients with the correct paperwork dramatically increased for a short space of time but this was not sustained (Fig. 5).

There was no significant change in patients’ awareness of the self-administration option. The proportion of patients who reported self-administering medicines and were recorded as doing so on the electronic prescribing system did not change and remained highly variable throughout the project.

Some patients made informal comments when completing questionnaires, after the leaflets and posters had been implemented, reporting that they were self-administering their medicines as a result of seeing the posters.

We carried out qualitative semi-structured interviews with four doctors, four nurses, four pharmacy staff and four therapists from the study wards. We had originally planned to carry out interviews with patients but as we had already evaluated the leaflets and posters with patients during the development process, our lay partners suggested that we focussed instead on healthcare professionals’ views. During the interviews, we asked healthcare professionals about their experiences of the QI project (reported in this section) and their views on self-administration and suggestions for the future (reported in next section below). Interviews were audio recorded, transcribed verbatim and analysed thematically following interviewees’ consent.

Due to rotations and staffing changes, a number of interviewees had not been on the relevant ward for the duration of the project, which meant that they were unable to comment on any changes noticed to self-administration during the project. Those who had been on the wards longer did not identify any major changes during the year. However, many reported that they were aware of the QI project and had seen the patient leaflets and posters or the posters in the doctors’ office.

## Lessons learned

### Raising healthcare professionals’ awareness

Our project suggests that raising healthcare professionals’ awareness of self-administration can greatly increase the proportion of patients who wish to self-administer who are actually able to do so. We found that it can be difficult to change processes on specific wards during a fixed time period. However, from the point when we began increasing our presence on the ward and having discussions with healthcare professionals about self-administration, we saw a significant and substantial increase in the proportion of patients who wished to self-administer who actually did so. It was difficult to assess the effect of the leaflets and posters aimed at patients due to the hospital being over capacity at the time they were implemented. However, interview data and informal discussions with patients suggested that leaflets and posters raised both healthcare professionals’ and patients’ awareness of self-administration, although quantitative data did not show a significant increase in patient awareness.

However, our findings also suggest that in order to sustain improvement, awareness needs to be a continuous rather than a one-off process. The improvements seen following the QI day, a one-off event, were not sustained. In contrast, those seen following more continuous awareness-raising on the study wards were sustained. In order to sustain improvement following the project, suggestions from healthcare professionals included incorporating training on self-administration into staff induction and education updates at the hospital organisation. Another recommendation was to make the self-administration assessment and process maps more accessible. These are currently incorporated into a lengthy policy document and healthcare professionals would prefer these to be available as separate documents. Many healthcare professionals, particularly doctors, junior nurses and therapists, indicated that they knew very little of the policy and that increasing training and making the key components more accessible would be helpful. We are working on embedding these recommendations at our hospital.

### Multi-disciplinary assessment process

Our findings suggested that rather than self-administration assessment being solely nurses’ responsibility, it should be multi-disciplinary. The majority of healthcare professionals interviewed were of the view that their profession could contribute to advising on patients’ suitability for self-administration but few were comfortable with their profession taking sole responsibility. All four doctors interviewed were willing, at least in theory, to take responsibility for carrying out initial assessment of patients for self-administration with others then having further input. A senior doctor was of the opinion that a potential barrier to this approach would be doctors generally having a low compliance rate with completing proformas. We were unable to facilitate doctors piloting self-administration assessments on our study wards on a larger scale during the study. However, we suspect that if the assessment could be better integrated into current electronic prescribing workflow in a more user friendly way, completion may increase and we are working on this within our organisation. Healthcare professionals were of the view that they did not have the time for an additional process but that most of the information required would be gathered as part of their current workflow and that it was a question of prompting them at the correct times to consider self-administration. Ideally, an automated process could be created where the electronic system draws together information from different records and identifies patients who may be suitable for self-administration. We are in discussion with our electronic prescribing software providers.

### Storage of medicines

Lack of an appropriate storage solution remained a barrier throughout the project. Either medicines were not being stored in accord with the hospital policy or patients were restricted to only being able to fully self-administer a restricted number of medicines. There is a need to develop a self-administration storage solution that can be accessed and opened by patients from their hospital bed. We are now working on this collaboratively with another organisation.

### Identifying patients as self-administering

We found that it was important for all healthcare professionals to know a patient was self-administering in order to keep this under review and ensure patients were informed of any changes to medication. To facilitate this, suitable systems need to be integrated into both policy and practice. Suggestions from interviewees were for both electronic and manual solutions; an example of the latter was to use coloured magnets on whiteboards.

While there is little published QI literature regarding self-administration, our findings build on previous research [9] that identified fragmentation of knowledge about medicines between healthcare professionals as well as barriers to self-administration. They are also corroborated by informal discussions we have had with other organisations that have carried out self-administration projects, which suggested that maintaining awareness though having a dedicated project lead and overcoming storage barriers [14] are key to success.

In summary, we were able to increase self-administration for patients who wanted it on selected wards in one hospital organisation. To sustain and spread this improvement further, further changes to hospital procedures are needed. For further improvement to take place, the development of a storage solution for self-administration is also likely to be required.

## Appendix 1

**Self-administration of medicines at St. Mary’s Hospital quality improvement project (SAMQI)**

We are carrying out a project to improve our systems for patients keeping and administering their own medicines in hospital if they would like to and are able to. We are interested in your views and experiences. The following questions should take <2 min to complete. There are no right or wrong answers, please choose the option which reflects your experiences.

Self-administration is when patients keep and administer their own medicines rather than nurses getting the medicines ready and bringing them to the patient.

**Table:** Patient questionnaire

	Yes	No	Uncertain
1. I am taking or being given medicines (other than through a drip) while in hospital. **If No go to question 7**	□	□	□
2. I know that some patients are able to keep and administer their own medicines in this hospital.	□	□	□
3. I have seen a leaflet about self-administration during this stay in hospital.	□	□	□
4. I have kept and administered my own medicines during this stay in hospital. **If No go to question 6**	□	□	□
5. The nurses know that I keep and administer my own medicines during this stay in hospital.	□	□	□
6. I would like to keep and administer my own medicines during this stay in hospital if I am able to.	□	□	□
7. I have kept and administered my own medication during previous visits to hospital.	□	□	□

Name of Hospital:

**Other data to be collected**

Is form for self-administration fully completed? Yes/No

Is signed off form for self-administration filed in the patients’ notes? Yes/No

Is patient recorded as self-administering on Cerner or paper drug chart? Yes/No (further details to be confirmed)
